# Factors Affecting the Occurrence of Out-of-Hospital Sudden Cardiac Arrest

**DOI:** 10.1155/2015/281364

**Published:** 2015-02-03

**Authors:** Izabella Uchmanowicz, Wiesław Bartkiewicz, Jarosław Sowizdraniuk, Joanna Rosińczuk

**Affiliations:** ^1^Department of Clinical Nursing, Faculty of Health Science, Wroclaw Medical University, 51-618 Wroclaw, Poland; ^2^Emergency Medical Service Station in Wroclaw, 54-203 Wroclaw, Poland; ^3^Department of Medical Emergency, Faculty of Health Science, Wroclaw Medical University, 51-618 Wroclaw, Poland

## Abstract

*Objective*. This paper aims to discover the risk factors for sudden cardiac arrest (out-of-hospital sudden cardiac arrest (OHSCA)) which significantly affect the decision about prioritizing emergency interventions before dispatching medical emergency teams, risk of deterioration of the patient's condition at the scene, and emergency procedures. *Methods*. A retrospective study taking into account the international classification of diseases ICD-10 based on an analysis of medical records of Emergency Medical Service in Wroclaw (Poland). *Results*. The main risk factor of OHSCA is coexistence of external cause leading to illness or death (ICD Group V-10) as well as the occurrence of diseases from the group of endocrine disorders (group E), in particular diabetes. The increase in the risk of OHSCA incidence is affected by nervous system diseases (group G), especially epilepsy of various etiologies, respiratory diseases (group J), mainly COPD, and bronchial asthma or mental and behavioral disorders (group F), with particular emphasis on the drugs issue. The procedure for receiving calls for Emergency Notification Centre does not take into account clinical risk factors for sudden cardiac arrest (SCA). *Conclusion*. Having knowledge of OHSCA risk factors can increase the efficiency of rescue operations from rapid assessment and provision of appropriate medical team, through effective performance of medical emergency treatment and prevention of SCA or finally reducing the costs.

## 1. Introduction

SCA (code I46 according to the International Statistical Classification of Diseases and Related Health Problems ICD-10) is a term relating to the cessation of cardiac mechanical activity with subsequent cessation of blood circulation. Secondary to these phenomena absent breathing occurs and then, in the absence of resuscitation, irreversible brain damage [1]. Data on the incidence of OHSCA in community are divergent due to the ambiguous definition of the disease, the sudden nature, and the fact that it usually occurs secondary to other diseases. It is estimated that SCA affects approximately 700 000 people in Europe annually [2]. According to the data collected by the American Heart Association from 2013, in the United States, there are around 359 000 cases of OHSCA [1]. This corresponds to about 11.6 cases of the condition per 10,000 U.S. citizens. Clinical data indicate that one in four OHSCA incidents was not preceded by clinical symptoms [1].

Mader et al. emphasize that the most common OHSCA mechanisms observed by paramedic teams are nonshockable rhythms (65.7%). Results clearly indicate that patients with initial shockable rhythms (VF/pulseless VT), making up 23.9% of the studied group, had a higher survival rate. Initial nonshockable rhythms (asystole/PEA) progressing to shockable rhythms in the course of CPR had no significant impact on the survival rate [3].

The risk factors for SCA can be divided into two groups: endogenous and exogenous factors. The first of these factors includes being nonmodifiable, independent of the patient factors, related to the characteristics of health and demographic aspects (age, sex, coronary artery disease, etc.) [2, 4–8]. Group of exogenous factors comprise factors largely dependent on the patient and modifiable factors (lifestyle, smoking, alcohol abuse, etc.) [1, 2, 9, 10].

Prompt intervention and cardiopulmonary resuscitation (CPR) are crucial for survival and minimizing neurological damages in patients with SCA [11]. The availability of rescue services, prevention of cardiac arrest, resuscitation undertaken by the witnesses (including the use of automated external defibrillators (AED)), and advanced care are major elements of the chain of survival [12]. The appropriate classification of the emergency call, giving it appropriate priority and identifying the risk of OHSCA incidence, will influence the increase in the survival rate and minimize the consequences of SCA and secondarily reduce the cost of patient care in a state of sudden health threat.

The aim of this study is to identify factors influencing the occurrence of OHSCA and in particular,

- identification of differences in the distribution of demographic factors,
- identification of differences in the distribution of comorbidities,
- identification of differences in classification of emergency interventions.

## 2. Methods

The analysis included the data referring to the interventions of medical emergency teams which took place between 1st January and 31st March 2013. Overall, in the analyzed period there were 26 219 interventions, including 245 (0.9%) visits to patients with diagnosis, in a medical card emergency operations, described as I46 according to ICD-10 classification meaning sudden cardiac arrest.

The first stage was to examine medical records of emergency operations based on an established diagnosis of I46 according to ICD-10. It allowed selecting a group of patients who experienced OHSCA and the second group who did not experience the above condition. Both groups were compared in terms of the severity of the potential risk factors of OHSCA incidence, which helped to identify three groups of risk factors:

- demographic factors:
  - patient's age, expressed as a continuous variable,
  - patient's age, expressed as a qualitative variable, with age limit 45 years for men and 55 for women,
  - sex;
- characteristic of emergency call:
  - illness,
  - medical emergency,
  - traffic accident,
  - accident at home,
  - accident at work,
  - accident at school,
  - accident in agriculture,
  - other accidents,
  - emergency service response code (1: emergency ambulance responding to a call up to 60 seconds, 2: up to 120 seconds, and 3: nonemergency response);
- clinical factors:
  - diagnosis of diseases from the major groups of ICD-10 classification (A to Z), or lack of thereof,
  - conditions comorbid to I46,
  - a number of conditions comorbid to I46.

Normality of distribution of continuous variables was assessed using the Shapiro-Wilk test, and their statistical characteristics were presented as arithmetic means, standard deviations, medians, interquartile ranges (IQR), and ranges. Statistical characteristics of qualitative variables were presented as number and percentage sequences. Mann-Whitney *U* test was used to compare the characteristics of continuous variables in the group of patients who experienced SCA and other patients. To compare the distributions of qualitative variables between the groups Pearson's chi-squared test was used.

The variables for which the above-mentioned comparisons between groups showed significant differences at *P* ≤ 0.05 were included in the logistic regression analysis. In the first step of this analysis, the strength and direction of the potential risk of OHSCA were studied in univariate analysis. The odds ratio (OR) of SCA incidence at patients with identified factor was calculated along with the 95% confidence interval. Variables that were characterized by a significance level *P* ≤ 0.05 were included in the multivariate logistic regression model, which aims to identify independent risk factors of OHSCA. All the above analyses were performed for the entire group and for subgroups of women and men. Calculations were done using the software Statistica 10 (StatSoft, USA), as the threshold of significance for all tests assuming the level of *P* ≤ 0.05.

## 3. Results

The mean age of patients with OHSCA was 63.5 years (IQR: 40–80 years) and did not differ significantly in comparison to the group of patients who did not develop OHSCA (*P* = 0.448). Among those who experienced OHSCA, there were 64% of patients at risk connected with their age. This percentage was not significantly different from the one observed in the subgroup of patients who show no evidence of OHSCA (*P* = 0.741).

The study showed a significantly higher percentage of women in the group of patients who experienced OHSCA (58.0%) than among other patients (*P* = 0.049). During the analysis of the causes of emergency calls no significant differences were reported, and higher priorities were assigned to interventions in patients with OHSCA.

Table 1 shows the distribution of the incidence frequency of comorbid diagnoses for given major ICD-10 groups among patients who experienced OHSCA and other patients. It was found that in the group with OHSCA patients were much more often diagnosed with coexisting diseases from a group of disorders of the endocrine and nutritional and metabolic diseases (group E according to ICD-10, *P* = 0.001) and the group of external causes of morbidity and decease (group V; *P* = 0.007). Moreover, patient with OHSCA often (on the borderline of statistical significance) was diagnosed also with a disease from the nervous system disease group (group G, *P* = 0.051).

Detailed analysis revealed that among the diagnoses from group E made in patients with OHSCA were type 2 diabetes mellitus (E11, *n* = 7), type 1 diabetes mellitus (E10, *n* = 4), and excessive loss of fluids (E86, *n* = 1). Among the diagnoses from the G group epilepsy were dominant (G40, *n* = 14) and abnormal roots of the spinal nerves and nerve plexus (G54, *n* = 2). There was no evidence to prove differences in the frequency of incidence of comorbid diagnoses. During the analysis, it was observed that a group of patients with OHSCA included significantly more patients with at least two comorbid diagnoses (*P* = 0.008).

Based on the above results of the comparisons, the univariate logistic regression analysis included the following variables:

- sex;
- incidence of coexisting diagnosis of the main groups V, E, and G ICD-10;
- the incidence of at least two comorbid diagnoses.

Univariate logistic regression results are shown in Table 2.

The most serious risk factor for OHSCA proved to be comorbid diagnosis of external cause of disease or death (group V of ICD-10), which tripled the frequency of incidence. Other significant risk factors for OHSCA turned out to be (starting with the most serious ones) coexisting diagnosis of endocrine disorders and nutritional and metabolic diseases (group E according to ICD-10, close to the 2.6-fold increased risk), the incidence of at least two comorbid diagnoses (1,65-fold increased risk), and female gender (almost 1.3-fold increase in risk). All the variables were included in the multivariate logistic regression model, whose characteristics are shown in Table 3.

It is worth noting that in the group of men with OHSCA an important risk factor turned out to be comorbid diagnosis of diseases from the group of disorders of the endocrine and nutritional and metabolic diseases (group E according to ICD-10), which increased nearly four times the frequency of this complication.

## 4. Discussion

Many studies have proven that the principal risk factors for OHSCA are cardiovascular conditions, including ischemic heart disease.

One example is the cohort study on 130 patients with a history of OHSCA. The researchers observed that ischemic heart disease had been a factor contributing to the cardiac arrest in as much as 55% [13].

In the own study, no significant influence of OHSCA risk factors related to cardiovascular conditions was observed. The disparity is due to the fact that the study was limited to a statistical analysis of data from paramedic records. Records kept by paramedic teams often only contain a selection of information, with no confirmed cause of the cardiac arrest (e.g., by autopsy results or laboratory tests).

It should also be noted that in some OHCA cases it is not possible to establish the patient's history and the cause of the cardiac arrest [14].

The present analysis, despite the above-mentioned differences, indicates the necessity of taking less obvious risk factors for cardiac arrest into consideration. This could significantly influence the survival rate of patients in emergency medical conditions.

This study indicates that significant independent risk factors for OHSCA are coexisting diagnosis of external causes of illness or death and the coexisting diagnosis of endocrine, nutritional, and metabolic diseases. The role of the conditions of both groups in the pathogenesis of SCA does not raise major doubts and has been confirmed in several studies.

The group of external causes of disease or death includes injuries as a result of traffic accidents. Literature data suggest that SCA is a common consequence of this type of event and is associated with a very poor prognosis. The low survival rate of traffic accident victims is the result of the occurrence of multiple organ injury, including damage to the central circulation system and intrathoracic organs [15–18].

Analysis of disorders of the second group indicates a higher risk of OHSCA in patients with diabetes type 2 more often than the type 1; diabetes, especially type 1, is a documented risk factor for SCA. A special role is attributed to the presence of macro- and microvascular diabetic coronary vessels, as well as neuropathy within the autonomic nervous system [19]. The above-mentioned changes are combined in many cases with the consequences of the metabolic syndrome, such as hypercholesterolemia, atherosclerosis, and obesity. Frequently, the condition negatively affected the prognosis, which is quite promising due to its atraumatic nature [20].

In addition to the diseases mentioned above in this study, there were also three other categories of diseases identified which appeared more frequently in patients with OHSCA. These were nervous diseases (group G according to the ICD-10), respiratory diseases (group J), and mental and behavioral disorders (group F).

The role of these apparently distant in etiological terms diseases in the pathogenesis of SCA could be easier to understand based on the results of a detailed analysis of comorbid conditions in patients with this disorder. Epilepsy (G40) accounts for the vast majority of the nervous system diseases occurring in patients with SCA. Incidence of disorders of the cardiovascular system, including SCA, in patients with epilepsy has been documented by a number of authors. One of the recently published pieces of evidence for the existence of this relationship is the results of a large Dutch study conducted by Amsterdam Resuscitation Studies (ARREST) evaluating the risk of SCA. The study demonstrated that the risk of SCA in patients with epilepsy is nearly three times higher than in those of the general population (OR = 2.9, 95% CI 1,1–8,0) [21]. The results of recent research suggest that the higher incidence of SCA in patients with epilepsy (sudden unexpected death in epilepsy, SUDEP) results from changes at the cellular level, including disorders of the serotonergic and adrenergic transduction [22]. The most common ailments of the respiratory system in patients experiencing SCA were chronic lung disease reservoir (J44), bronchial asthma (J45), and other states of asthma (J46). The role of these chronic diseases in the etiopathogenesis of heart failure does not raise any doubts [23]. Several authors have shown that both chronic obstructive pulmonary disease and asthma are associated with progressive bronchial remodeling, manifested by bronchial wall thickening and a reduction in their lumen. Apart from hypoxia, which may lead to abnormal autonomic cardiovascular control mechanisms, it may also result in changes in pulmonary hemodynamics and heart failure [11, 24].

In the group of mental and behavioral disorders, disorders related to alcohol predominate (F10). The direct consequence is alcohol cardiomyopathy, resulting in severe myocardial infarction. Alcohol abuse may be an indirect cause of heart failure through an adverse effect on hepatic metabolism of lipids and increased risk of coronary atherosclerosis [25]. Finally, it is worth noting that there are documented SCA in alcoholics in the process of alcohol rehabilitation [26].

In addition to clinical variables, this analysis has identified yet another independent risk factor for OHSCA, which is the female sex. This observation seems surprising, given that according to the literature the male gender is more susceptible to this condition [2]. The explanation for this unexpected result comes from a detailed analysis of risk criteria in the subgroup of women and men. They showed that men and women participating in this study represented different comorbidity profiles. Among women, there were many more victims of traffic accidents than men. It is very probable that this fact decided that the female gender has proven to be a significant independent risk factor for SCA in the group. Uneven distribution of comorbidities in the analyzed sample should be considered as one of the potentially disturbing factors and limitations of this study.

In addition to confirming, in the well-documented literature, a role of a number of comorbidities in the etiopathogenesis of SCA, the results of this study provide numerous presumptions for the functioning of the State Emergency Medical Services. None of the analyzed characteristics of the call acceptance has proved to be associated with the incidence frequency of OHSCA in a studied group. However, many studies have proven conclusively that the key factor determining the prognosis for patients with SCA is immediate emergency response [1, 11]. An important role in this process should play a dispatcher at Emergency Centre assessing the risk associated with the call and giving it the right priority to provide proper service. The results of this study indicate that this system is not fully effective and suggest the need for verification and refinement.

Potential limitations of a given study may result from

- uneven distribution of medical conditions coexisting in a group of women and men;
- the analysis focusing on selected groups of patients and the emergency risk profile deviating significantly from the characteristics of the general population. This limitation seems to be a potential cause why this study failed to prove the role of cardiovascular diseases as the main risk factor of SCA;
- a retrospective nature of the analysis, the incompleteness of data, and different coding of the same diagnoses by different people. The potential effect of this disturbing factor has been neutralized by a large number of the studied sample population.

## 5. Conclusions

Based on the results of this analysis the following conclusions can be made regarding the risk factors of OHSCA.

- An essential element is the presence of an external cause of the patient's illness or death (group V of ICD-10), or concomitant diseases from a group of disorders of the endocrine and nutritional and metabolic diseases (group E according to ICD-10), particularly diabetes.
- Higher risk of SCA may be indicated by coexisting nervous system diseases (group G according to the ICD-10 and in particular epilepsy), respiratory system diseases (group J, in particular chronic obstructive pulmonary disease and asthma), and mental and behavioral disorders (group F, especially of alcoholic etiology).
- The analysis of emergency calls and their codification should include the above-mentioned clinical conditions, which do not exist at the moment.

## Figures and Tables

**Table 1:** Distribution of the incidence of comorbid conditions from given major categories of the ICD-10 classification in patients who developed OHSCA and other patients.

Group ICD-10	OHSCA		Non-OHSCA		*P* value
	*n*	%	*N*	%	
A	2	0,8	72	0,3	0,113
B	0	0,0	19	0,1	0,672
C	3	1,2	215	0,8	0,496
D	0	0,0	52	0,2	0,483
E	12	4,9	509	2,0	0,001
F	16	6,5	1233	4,8	0,192
G	16	6,5	1053	4,1	0,051
H	0	0,0	29	0,1	0,601
I	47	19,2	4501	17,3	0,445
J	16	6,5	1226	4,7	0,184
K	1	0,4	499	1,9	0,085
L	0	0,0	75	0,3	0,400
M	7	2,9	482	1,9	0,249
N	1	0,4	358	1,4	0,193
O	1	0,4	145	0,6	0,753
P	0	0,0	12	0,1	0,737
Q	0	0,0	2	0,0	0,891
R	82	33,5	10121	39,0	0,079
S	33	13,5	3268	12,6	0,677
T	6	2,4	802	3,1	0,565
V	5	2,0	168	0,7	0,007
W	3	1,2	150	0,6	0,186
X	0	0,0	110	0,4	0,307
Y	0	0,0	321	1,2	0,080
Z	10	4,1	976	3,8	0,791

Source: own study.

**Table 2:** The values of the OR of OHSCA in patients at potential risk factors for this complication, as defined in the univariate logistic regression analysis.

Parameter	OR	95% CI	*P* value
Female gender	1,29	1,00–1,66	0,048
Group E ICD-10	2,58	1,43–4,63	0,002
Group V ICD-10	3,20	1,30–7,86	0,011
Group G ICD-10	1,65	0,99–2,76	0,054
Min. 2 diagnoses	1,65	1,14–2,39	0,008

Source: own study.

**Table 3:** The values of the OR of SCA in patients at significant risk for this complication, as defined in the multivariate logistic regression analysis.

Parameter	OR	95% CI	*P* value
Group V ICD-10	3,04	1,23–7,53	0,016
Group E ICD-10	2,28	1,25–4,18	0,008
Min. 2 diagnoses	1,46	1,00–2,15	0,052
Female gender	1,30	1,01–1,67	0,044

Source: own study.

## References

[1] Go A. S., Mozaffarian D., Roger V. L. (2014). Heart disease and stroke statistics—2014 update: a report from the American Heart Association. *Circulation*.

[2] Engdahl J., Holmberg M., Karlson B. W., Luepker R., Herlitz J. (2002). The epidemiology of out-of-hospital ‘sudden’ cardiac arrest. *Resuscitation*.

[3] Mader T. J., Nathanson B. H., Millay S., Coute R. A., Clapp M., McNally B. (2012). Out-of-hospital cardiac arrest outcomes stratified by rhythm analysis. *Resuscitation*.

[4] Merghani A., Sharma S. (2012). Identifying patients at risk of sudden arrhythmic death. *Practitioner*.

[5] Gräsner J.-T., Bossaert L. (2013). Epidemiology and management of cardiac arrest: what registries are revealing. *Best Practice and Research: Clinical Anaesthesiology*.

[6] Galea S., Blaney S., Nandi A. (2007). Explaining racial disparities in incidence of and survival from out-of-hospital cardiac arrest. *The American Journal of Epidemiology*.

[7] Rea T. D., Pearce R. M., Raghunathan T. E. (2004). Incidence of out-of-hospital cardiac arrest. *The American Journal of Cardiology*.

[8] Bennett M. T., Sanatani S., Chakrabarti S., Deyell M. W., Krahn A. D. (2013). Assessment of genetic causes of cardiac arrest. *Canadian Journal of Cardiology*.

[9] Chiuve S. E., Fung T. T., Rexrode K. M. (2011). Adherence to a low-risk, healthy lifestyle and risk of sudden cardiac death among women. *JAMA—Journal of the American Medical Association*.

[10] Thun M. J., Peto R., Lopez A. D. (1997). Alcohol consumption and mortality among middle-aged and elderly U.S. adults. *The New England Journal of Medicine*.

[11] Nolan J. P., Soar J., Zideman D. A. (2010). European Resuscitation Council guidelines for resuscitation 2010. *Resuscitation*.

[12] Kubler A., Mysiak A. (2005). *Choroba Poresuscytacyjna*.

[13] Adrie C., Cariou A., Mourvillier B. (2006). Predicting survival with good neurological recovery at hospital admission after successful resuscitation of out-of-hospital cardiac arrest: the OHCA score. *European Heart Journal*.

[14] Giovannetti O., Dumas F., Lemiale V. (2010). 338 Causes and hospital mortality in a large cohort of out-of-hospital cardiac arrest. *Archives of Cardiovascular Diseases Supplements*.

[15] Fisher B., Worthen M. (1999). Cardiac arrest induced by blunt trauma in children. *Pediatric Emergency Care*.

[16] Rosemurgy A. S., Norris P. A., Olson S. M. (1993). Prehospital traumatic cardiac arrest: the cost of futility. *Journal of Trauma*.

[17] Cook T. M., Gupta K. (2007). Emergency thoracotomy after cardiac arrest from blunt trauma is not always futile. *Resuscitation*.

[18] Crewdson K., Lockey D., Davies G. (2007). Outcome from paediatric cardiac arrest associated with trauma. *Resuscitation*.

[19] Siscovick D. S., Sotoodehnia N., Rea T. D., Raghunathan T. E., Jouven X., Lemaitre R. N. (2010). Type 2 diabetes mellitus and the risk of sudden cardiac arrest in the community. *Reviews in Endocrine and Metabolic Disorders*.

[20] Larsson M., Thorén A.-B., Herlitz J. (2005). A history of diabetes is associated with an adverse outcome among patients admitted to hospital alive after an out-of-hospital cardiac arrest. *Resuscitation*.

[21] Bardai A., Lamberts R. J., Blom M. T. (2012). Epilepsy is a risk factor for sudden cardiac arrest in the general population. *PLoS ONE*.

[22] Massey C. A., Sowers L. P., Dlouhy B. J., Richerson G. B. (2014). Mechanisms of sudden unexpected death in epilepsy: the pathway to prevention. *Nature Reviews Neurology*.

[23] Warnier M. J., Blom M. T., Bardai A. (2013). Increased risk of sudden cardiac arrest in obstructive pulmonary disease: a case-control study. *PLoS ONE*.

[24] Soar J., Perkins G. D., Abbas G. (2010). European Resuscitation Council Guidelines for Resuscitation 2010, Section 8. Cardiac arrest in special circumstances: electrolyte abnormalities, poisoning, drowning, accidental hypothermia, hyperthermia, asthma, anaphylaxis, cardiac surgery, trauma, pregnancy, electrocution. *Resuscitation*.

[25] Walker R. K., Cousins V. M., Umoh N. A. (2013). The good, the bad, and the ugly with alcohol use and abuse on the heart. *Alcoholism: Clinical and Experimental Research*.

[26] Littmann L., Monroe M. H., Anderson J. D. (2003). Images in cardiology: ‘All shook up’. *Clinical Cardiology*.

